# Supplementary figures and images for: Syndromic surveillance: A key component of population health monitoring during the first wave of the COVID-19 outbreak in France, February-June 2020
Source: PLoS One. 2022 Feb 10;17(2):e0260150. doi: 10.1371/journal.pone.0260150 (PMC8830636; doi:10.1371/journal.pone.0260150)

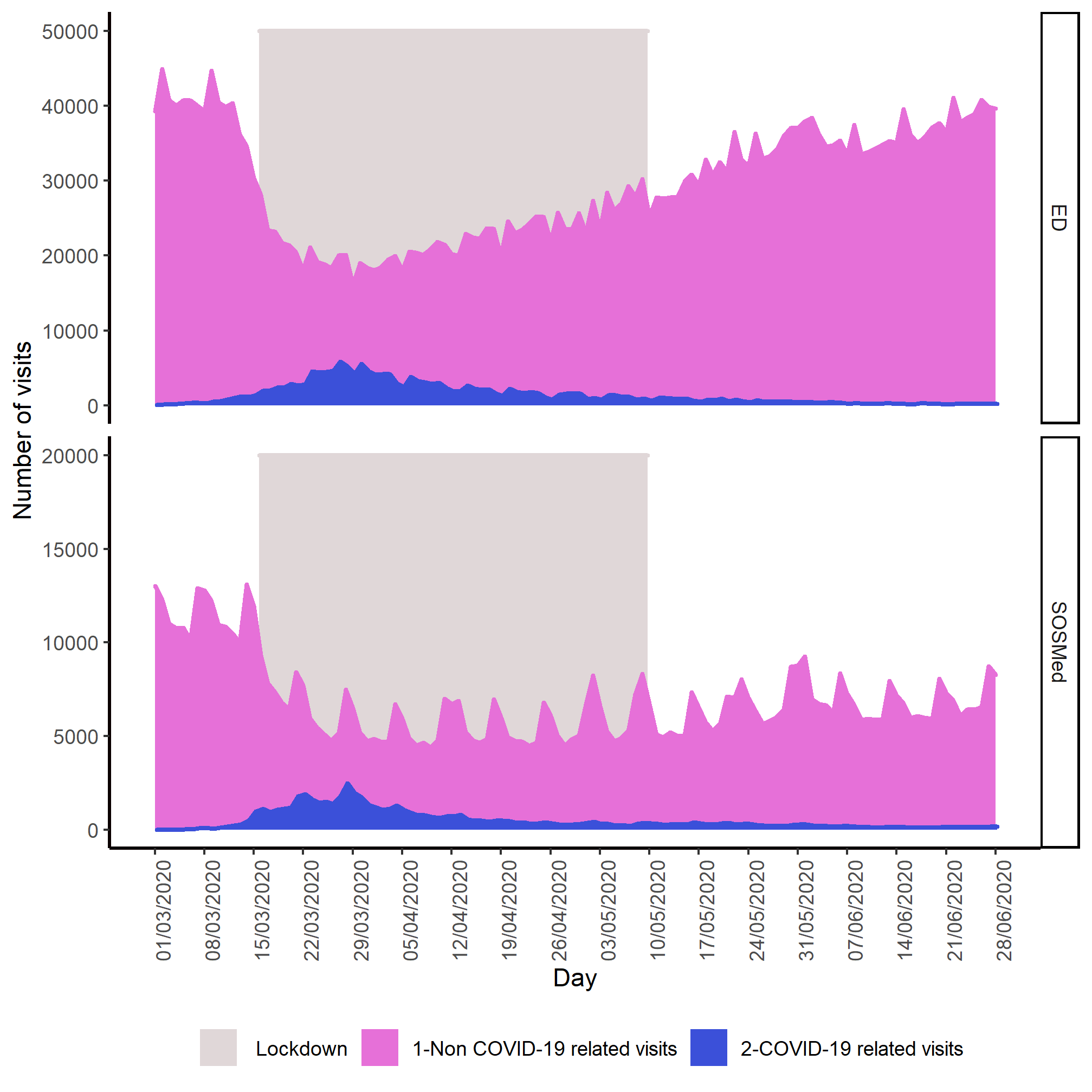

Supplement: S1 Fig — (TIF) [file pone.0260150.s001.tif]
