# Supplementary material for: Syndromic surveillance: A key component of population health monitoring during the first wave of the COVID-19 outbreak in France, February-June 2020
Source: PLoS One. 2022 Feb 10;17(2):e0260150. doi: 10.1371/journal.pone.0260150 (PMC8830636; doi:10.1371/journal.pone.0260150)
Supplement: S1 Table — (DOCX) [file pone.0260150.s002.docx]

**S1 Table. Diagnoses associated with COVID-19-related emergency department visits from 17 February to 28 June 2020.**

|  | Number of use  (N) | Proportion of visits among overall associated diagnoses (%) |
| --- | --- | --- |
| Total number of associated diagnoses | 18,939 | - |
|  |  |  |
| Pulmonary infectious disease (pneumonia, bronchitis, bronchiolitis) | 2,073 | 10.95 |
| Cardiopathy (high blood pressure, cardiac failure, pulmonary embolism) | 1,543 | 8.15 |
| COVID-19 | 1,465 | 7.74 |
| Dyspnoea | 1,247 | 6.58 |
| Cough | 926 | 4.89 |
| Chronic pulmonary disease (asthma, emphysema, cancer) | 881 | 4.65 |
| Unknown fever | 833 | 4.40 |
| Asthenia and faintness | 696 | 3.67 |
| Thoracic pain | 689 | 3.64 |
| Person with feared health complaint | 593 | 3.13 |
| Isolation | 533 | 2.81 |
| Mental health disorder | 423 | 2.23 |
| ENT disease | 375 | 1.98 |
| Respiratory failure | 348 | 1.84 |
| Hydroelectrolytic disorders | 307 | 1.62 |
| Kidney failure | 296 | 1.56 |
| Gastrointestinal disorder | 285 | 1.50 |
| Diabetes | 264 | 1.39 |
| Stroke and neurological disorders | 235 | 1.24 |
| Influenza-like illness | 126 | 0.67 |
| Viral infection | 116 | 0.61 |
| Septicemia | 110 | 0.58 |
