# Supplementary material for: Syndromic surveillance: A key component of population health monitoring during the first wave of the COVID-19 outbreak in France, February-June 2020
Source: PLoS One. 2022 Feb 10;17(2):e0260150. doi: 10.1371/journal.pone.0260150 (PMC8830636; doi:10.1371/journal.pone.0260150)
Supplement: S2 Table — (DOCX) [file pone.0260150.s003.docx]

**S2 Table. Diagnoses associated with COVID-19-related SOSMed visits from 17 February to 28 June 2020**

|  | Number of visits (N) | Proportion of visits among overall associated diagnoses (%) |
| --- | --- | --- |
| Total number of associated diagnoses | 1,882 | - |
|  | | |
| ENT (rhinopharyngitis, angina, tracheitis, sinusitis, otitis) | 454 | 24.1 |
| Gastroenteritis | 168 | 8.9 |
| Acute bronchitis | 137 | 7.3 |
| Acute pneumonia | 124 | 6.6 |
| Anxiety | 111 | 5.9 |
| Influenza-like illness | 96 | 5.1 |
| Isolated fever | 61 | 3.2 |
| Diarrhoea | 49 | 2.6 |
| Urinary infection | 47 | 2.5 |
| Allergy | 46 | 2.4 |
| Thoracic pain | 43 | 2.3 |
| Acute respiratory failure | 38 | 2.0 |
| Asthma | 32 | 1.7 |
| Viral infection | 19 | 1.0 |
| Headaches | 16 | 0.9 |
| Death | 16 | 0.9 |
| Impaired general state | 14 | 0.7 |
