# Supplementary material for: Syndromic surveillance: A key component of population health monitoring during the first wave of the COVID-19 outbreak in France, February-June 2020
Source: PLoS One. 2022 Feb 10;17(2):e0260150. doi: 10.1371/journal.pone.0260150 (PMC8830636; doi:10.1371/journal.pone.0260150)
Supplement: S3 Table — (DOCX) [file pone.0260150.s004.docx]

**S3 Table. Most common complaints in COVID-19-related SOSMed visits from 17 February to 28 June 2020**

|  | Number of visits  (N) | Proportion of visits among overall associated diagnoses (%) |
| --- | --- | --- |
| Total number of chief complaints | 114,767 | - |
|  | | |
| Fever and sweat | 20,625 | 18.0 |
| Cough | 18,349 | 16.0 |
| ENT (sore throat, cold) | 13,004 | 11.3 |
| Gastrointestinal disorder (diarrhoea, vomiting, abdominal pain) | 10,734 | 9.4 |
| Headaches | 9,408 | 8.2 |
| Muscular pain | 7,553 | 6.6 |
| Dyspnoea | 6,606 | 5.8 |
| Asthenia and faintness | 6,077 | 5.3 |
| Thoracic pain | 2,69 | 2.3 |
| Pneumopathy | 1,843 | 1.6 |
| Dizziness | 1,79 | 1.6 |
| Influenza like illness | 1,44 | 1.3 |
| Suspected COVID-19 | 1,37 | 1.2 |
| Tremor | 1,246 | 1.1 |
| Back and cervical pain | 1,209 | 1.1 |
| Anxiety | 778 | 0.7 |
| Palpitation | 582 | 0.5 |
| Bronchitis | 502 | 0.4 |
| Impaired general state | 322 | 0.3 |
| Skin eruption | 310 | 0.3 |
