# Supplementary material for: Syndromic surveillance: A key component of population health monitoring during the first wave of the COVID-19 outbreak in France, February-June 2020
Source: PLoS One. 2022 Feb 10;17(2):e0260150. doi: 10.1371/journal.pone.0260150 (PMC8830636; doi:10.1371/journal.pone.0260150)
Supplement: Attachment — Submitted filename: Response_to_Reviewers.docx [file pone.0260150.s006.docx]

**Answers to reviewers**

We thank the reviewers for the time spent on this new review and for the comments. We have taken into account all the recommendations. Detail of the answers to the comments are below (in bold).

**References review:**

Comments: Please review your reference list to ensure that it is complete and correct. If you have cited papers that have been retracted, please include the rationale for doing so in the manuscript text, or remove these references and replace them with relevant current references. Any changes to the reference list should be mentioned in the rebuttal letter that accompanies your revised manuscript. If you need to cite a retracted article, indicate the article’s retracted status in the References list and also include a citation and full reference for the retraction notice.

**As recommended by journal requirements, we used Vancouver style trough Endnote software for formatting our references.**

**We also made some modifications in our list of references between the 1^st^ and 2^nd^ version of manuscript submitted. Please find below the changes made:**

- **For reference 1, the website link was updated (1.	WHO. Novel coronavirus China. Geneva: World Health Organization; 2020 [Available from: <https://www.who.int/emergencies/disease-outbreak-news/item/2020-DON233>.);**
- **For reference 5, the website link was updated. The current link direct to SpFrance COVID-19 web page which is regularly updated (5.	France Sp. Coronavirus (COVID-19) - Santé publique France 2021 [Available from:** [**https://www.santepubliquefrance.fr/dossiers/coronavirus-covid-19**](https://www.santepubliquefrance.fr/dossiers/coronavirus-covid-19)**)**
- **We have retracted the following reference :	France Sp. Point épidémiologique hebdomadaire national [Available from:** [**https://www.santepubliquefrance.fr/maladies-et-traumatismes/maladies-et-infections-respiratoires/infection-a-coronavirus/documents/bulletin-national/covid-19-point-epidemiologique-du-27-aout-2020**](https://www.santepubliquefrance.fr/maladies-et-traumatismes/maladies-et-infections-respiratoires/infection-a-coronavirus/documents/bulletin-national/covid-19-point-epidemiologique-du-27-aout-2020)**.**

**This reference was cited to highlight surveillance systems used to monitor COVID-19 outbreak in France. Instead of having a specific reference, pointing to a single bulletin, we preferred to cite the reference 5.	France Sp. Coronavirus (COVID-19) - Santé publique France 2021 [Available from: <https://www.santepubliquefrance.fr/dossiers/coronavirus-covid-19>, which is SpFrance web page dedicated to COVID-19 with various information (data, statistics, bulletins) regularly updated.**

- **Reference 13 was added to highlight utilization of other data used to monitor outbreak (laboratory-confirmed tests, hospital and intensive care admissions for COVID-19, COVID-19 related deaths) and the reproduction number R of the epidemic.**

**(13.	France Sp. COVID-19 : point épidémiologique du 30 juillet 2020 France: Sante publique France; [updated 30 july 2020. Available from: <https://www.santepubliquefrance.fr/maladies-et-traumatismes/maladies-et-infections-respiratoires/infection-a-coronavirus/documents/bulletin-national/covid-19-point-epidemiologique-du-30-juillet-2020>.).**

- **References 14 to 16 were added to emphasize our discussion on sex comparison regarding COVID-19.**

**14.	Kaeuffer C, Le Hyaric C, Fabacher T, Mootien J, Dervieux B, Ruch Y, et al. Clinical characteristics and risk factors associated with severe COVID-19: prospective analysis of 1,045 hospitalised cases in North-Eastern France, March 2020. Eurosurveillance. 2020;25(48):2000895.**

**15.	surveillance Wgft, Spain coC-i. The first wave of the COVID-19 pandemic in Spain: characterisation of cases and risk factors for severe outcomes, as at 27 April 2020. Eurosurveillance. 2020;25(50):2001431.**

**16.	Stokes EK, Zambrano LD, Anderson KN, Marder EP, Raz KM, El Burai Felix S, et al. Coronavirus Disease 2019 Case Surveillance - United States, January 22-May 30, 2020. MMWR Morb Mortal Wkly Rep. 2020;69(24):759-65.**

A. **Comments to the Author**

Reviewer #1: The revision is responsive and has addressed the reviewer’s previous critiques.

Thank you very much.

**Minor comments:**

1. The figures read blurred in the PDF, making it difficult to see the details.

**Sorry for this blurred figures. The figures have now been exported in tiff format as recommended in the guidelines and should be in a better quality. We also used PACE tools to meet requirement journal’s for figure 3.**

2. The stacked area charts of non-COVID and COVID-related visits (in the response letter) look informative. It could be included as supplemental results if the authors do not feel like putting them in the main manuscript.

**We added in supporting information the stacked area charts of non-COVID and COVID-related visits with the following captions: S1 Fig. Number of COVID-19 and non-COVID-19 related visits in ED and SOSMed associations, from 17 February to 28 June 2020 in France**

3. The use of English could benefit from another round of proofread by a native speaker.

**An English speaker have proofread the current manuscript.**
